# Beyond the Needle: A Comparative Evaluation of Silk Sutures and Cyanoacrylate for Periodontal Flap Closure

**DOI:** 10.7759/cureus.56604

**Published:** 2024-03-20

**Authors:** Jannat Gautam, Anchal Sood, Swantika Chaudhry, Simran Ghumman, Anshul Chopra

**Affiliations:** 1 Department of Periodontology, Baba Jaswant Singh Dental College Hospital & Research Institute, Ludhiana, IND; 2 Department of Periodontology, Clove Dental, New Delhi, IND; 3 Department of Oral and Maxillofacial Surgery, Christian Dental College and Hospital, Ludhiana, IND

**Keywords:** needle, periodontology, cyanoacrylate, closure, flap

## Abstract

Introduction

As the incidence of periodontal diseases continues to surge, there is a concurrent elevation in the demand for periodontal treatment. Periodontal surgical therapy is done to control and eliminate disease activity. Conventionally, silk sutures have been considered the gold standard for post-operative flap closure that leads to biofilm accumulation and tissue trauma. Cyanoacrylates are alternate options to avoid the limitations.

Objective

The objective of the study was to assess clinical outcomes by comparing the healing after periodontal flap surgery when secured with 3-0 braided silk suture versus cyanoacrylate.

Methodology

Twenty surgical sites from 10 patients with moderate to severe periodontitis were selected and randomly divided into two groups after phase-1 therapy: the test group (isoamyl 2-cyanoacrylate) and the control group (3-0 silk braided suture). Post-operative wound healing, pain assessment using a verbal rating scale (VRS), and analgesic tablets taken were evaluated on the third, fifth, seventh, and 14th days. Statistical analysis was done using the ANOVA test with the post-hoc Bonferroni test.

Results

There was no statistically significant difference between the VRS and wound healing index at different levels of intervals during intergroup comparison, but the number of analgesics consumed post-operatively was less in the test group as compared to the control group.

Conclusion

The present study concluded that isoamyl 2-cyanoacrylate can be used as an alternative to conventional silk sutures as it decreases post-operative pain and discomfort.

## Introduction

In the continuum of dental practice, dental professionals have centered their efforts on delivering comprehensive oral health care, with a primary emphasis on ensuring patient comfort. The WHO Global Health Status Report 2022 stated that severe periodontal diseases are estimated to affect around 19% of the global adult population, representing over one billion cases worldwide [1]. As the incidence of cases continues to surge, there is a concurrent elevation in the demand for periodontal treatment.

Since the beginning of time, surgical sutures have been used to close wounds without difficulty. A variety of materials, including human hair and the silk sutures used today, have been tried in this process. However, even with advanced suture materials and techniques, there are times when the wound closure is not as successful as desired and can result in complications such as fistulation and granuloma formation [2]. Periodontal surgical therapy is done with the objective of eliminating and controling periodontal disease activity [2]. The predominant method for wound closure involves the application of sutures. Sutures are used to approximate the wound margins, ensuring wound stabilization and primary healing. Conventionally silk sutures have been considered a gold standard for post-operative flap closure [3]. With prolonged practical utilization, certain shortcomings have been observed in the use of sutures such as slicing through the parenchymal and inflammatory tissues during suturing, black suture materials that are twisted or braided to exhibit capillary action, etc. It is a technique-sensitive procedure that takes a lot of time during the surgical procedure [4].

Braided silk sutures also exhibit the characteristic property of colonization of biofilms due to the ‘wicking’ action that increases the bacterial infiltration of tissue. Additionally, this property serves as a reservoir for secondary infections [5]. There were also increased chances of tissue penetration trauma that might lead to tissue necrosis and delayed healing. To overcome these limitations alternate options for wound closure were explored. Tissue bioadhesives were studied widely in the 19th century as a suitable alternate option for post-operative flap approximation. The desirable characteristics of tissue bioadhesives were considered to be non-toxic, non-allergic, non-carcinogenic, absorbable, degradable in due time, and non-irritating locally and systemically [6]. One such substance that closely matches the criteria is cyanoacrylate tissue bioadhesive. In 1949, Ardis made the discovery and synthesis of chemical adhesives. When Coover eventually reported on their adhesive properties, they were used on humans for the first time in 1959 [7,8]. The use of bioadhesives tends to avoid tissue penetration trauma [7]. The objective of the current study was to assess the clinical outcomes by comparing the healing process after periodontal flap surgery when secured with 3-0 braided silk suture versus cyanoacrylate bioadhesive.

## Materials and methods

The comparative clinical study was conducted at the Department of Periodontology and Oral Implantology, Baba Jaswant Singh Dental College and Hospital, Ludhiana, Punjab, from August 2021 to July 2022. The ethical approval for the study was taken from the institute, with institutional review board number (IRB) IEC/BJS/2021/RA-02. A total of 20 surgical sites were selected from 10 patients who met the inclusion and exclusion criteria. The selection of subjects was regardless of gender, caste, religion, or socio-economic status.

Inclusion criteria for the study were as follows: Phase-I-treated patients, patients with periodontal probing depths of 5 mm or deeper after Phase-I therapy indicating a need for periodontal flap surgery, systemically healthy patients confirmed through blood investigation, and those without other active painful conditions such as an abscess or tooth fracture. Exclusion criteria included pregnant or lactating mothers, patients with a history of smoking or tobacco consumption, and systemically unhealthy patients with poor oral hygiene.

After screening and selection based on the criteria, 20 surgical sites were randomly allocated to two different groups: Group I, utilizing braided silk 3-0 sutures (Ethicon Mersilk, Johnson & Johnson Pvt Ltd), and Group II, using isoamyl-2 cyanoacrylate (Amcrylate, Concord Drugs Ltd., India) as a tissue bioadhesive. The surgical procedures were performed under local anesthesia (2% lignocaine with adrenaline, Lignocad Adr 1:2,00,000). A number 15 blade was used for intrasulcular incision, followed by elevation of a full-thickness mucoperiosteal flap and debridement/root planning. Suturing was performed using either braided silk 3-0 sutures, and a surgeon's knot was used as a knot variant or isoamyl-2 cyanoacrylate, depending on the group.

Parameters evaluated included the following: (a) wound healing index, assessed using a scoring system ranging from very poor to excellent based on tissue color, response to palpation, and suppuration [6]; b) verbal rating scale for pain assessment, ranging from none to very severe [9]; and c) the number of analgesics taken post-operatively. Data were evaluated on the third, fifth, seventh, and 14th days post-operatively, with digital photographic records taken at each visit. Statistical analysis was conducted using Statistical Product and Service Solutions (SPSS, version 21; IBM SPSS Statistics for Windows, Armonk, NY), employing independent unpaired t-tests for intergroup comparison and one-way analysis of variance (ANOVA) with post-hoc Bonferroni for intragroup comparison within both groups. p-value was considered significant at <0.05.

## Results

The clinical data from 20 surgical sites were recorded for the comparative analysis of suture and isoamyl-2 cyanoacrylate groups. The intragroup comparison revealed that the mean score of the verbal rating scale and wound healing index differ significantly at different intervals of time (p < 0.001). For Group I, the verbal rating scale worked out to 2.80 on day three, which came down to 1.50 on day five and further fell to 0.70 and 0.20 on days seven and 14, respectively (Table 1).

**Table 1:** Intragroup comparison of the verbal rating scale (Group I) Group I: Utilizing braided silk 3-0 sutures Verbal rating scale for pain assessment, ranging from none to very severe. N: Number of patients, SD: Standard deviation, SE: Standard error Statistical test used: Analysis of variance (ANOVA) where a p-value was considered significant if p < 0.05.

Follow-up evaluation	Verbal Rating Scale			
	N	Mean	±SD	SE
Day 3	10	2.80	1.23	0.39
Day 5	10	1.50	0.85	0.27
Day 7	10	0.70	0.82	0.26
Day 14	10	0.20	0.42	0.13
F-ratio	16.662			
p-value	<0.001			

On the contrary, the wound healing index differs significantly at different intervals. It worked out to 1.80 on day three, which came up to 2.40 on day five and further increased to 3.20 and 3.80 on days seven and 14. It improved significantly on day 14 (Table 2).

**Table 2:** Intragroup comparison of the wound healing index (Group I) Group I: Utilizing braided silk 3-0 sutures Wound healing index assessed using a scoring system ranging from very poor to excellent based on tissue color, response to palpation, and suppuration. N: Number of patients, SD: Standard deviation, SE: Standard error Statistical test used: Analysis of variance (ANOVA) where a p-value was considered significant if p < 0.05.

Follow-up evaluation	Wound Healing Index			
	N	Mean	±SD	SE
Day 3	10	1.80	0.63	0.20
Day 5	10	2.40	0.52	0.16
Day 7	10	3.20	0.42	0.13
Day 14	10	3.80	0.63	0.20
F-ratio	24.857			
p-value	<0.001			

For Group II, the mean score of the verbal rating scale differs significantly at different intervals of time. The verbal rating scale worked out to 2.90 on day three, which came down to 0.80 on day five and further fell to 0.30 and 0.10 on days seven and 14, respectively (Table 3).

**Table 3:** Intragroup comparison of the verbal rating scale (Group II) Group II: Using isoamyl-2 cyanoacrylate Verbal rating scale for pain assessment, ranging from none to very severe. N: Number of patients, SD: Standard deviation, SE: Standard error Statistical test used: Analysis of variance (ANOVA) where a p-value was considered significant if p < 0.05.

Follow-up evaluation	Verbal Rating Scale			
	N	Mean	±SD	SE
Day 3	10	2.90	1.37	0.43
Day 5	10	0.80	1.03	0.33
Day 7	10	0.30	0.48	0.15
Day 14	10	0.10	0.32	0.10
F-ratio	20.125			
p-value	<0.001			

The mean score of wound healing index increased from 2.30 to 4.20 from day three to day 14, respectively (Table 4).

**Table 4:** Intragroup comparison of the wound healing index (Group II) Group II: Using isoamyl-2 cyanoacrylate Wound healing index, assessed using a scoring system ranging from very poor to excellent based on tissue color, response to palpation, and suppuration. N: Number of patients, SD: Standard deviation, SE: Standard error Statistical test used: Analysis of variance (ANOVA) where a p-value was considered significant if p < 0.05.

Follow-up evaluation	Wound Healing Index			
	N	Mean	±SD	SE
Day 3	10	2.30	0.67	0.21
Day 5	10	2.80	0.79	0.25
Day 7	10	3.40	0.52	0.16
Day 14	10	4.20	0.63	0.20
F-ratio	15.344			
p-value	<0.001			

There was no statistically significant difference between the verbal rating scale and wound healing index at different levels of intervals during the intergroup comparison (Tables 5-6).

**Table 5:** Intergroup comparison of the verbal rating scale Group I: Utilizing braided silk 3-0 sutures Group II: Using isoamyl-2 cyanoacrylate Verbal rating scale for pain assessment, ranging from none to very severe NS- stands for non-significant, i.e., p > 0.05 N: Number of patients, SD: Standard deviation, SE: Standard error A p-value was considered significant if p < 0.05.

Days (Follow-up)	Group I				Group II				Unpaired t-test	p-value	Level of significance
	N	Mean	±SD	SE	N	Mean	±SD	SE			
Day 3	10	2.80	1.23	0.39	10	2.90	1.37	0.43	-0.172	0.866	NS
Day 5	10	1.50	0.85	0.27	10	0.80	1.03	0.33	1.655	0.115	NS
Day 7	10	0.70	0.82	0.26	10	0.30	0.48	0.15	1.325	0.202	NS
Day 14	10	0.20	0.42	0.13	10	0.10	0.32	0.10	0.600	0.556	NS

**Table 6:** Intergroup comparison of the wound healing index Group I: Utilizing braided silk 3-0 sutures Group II: Using isoamyl-2 cyanoacrylate Wound healing index, assessed using a scoring system ranging from very poor to excellent based on tissue color, response to palpation, and suppuration NS- stands for non-significant, i.e., p > 0.05 N: Number of patients, SD: Standard deviation, SE: Standard error A p-value was considered significant if p < 0.05.

Days (Follow-up)	Group I				Group II				Unpaired t-test	p-value	Level of significance
	N	Mean	±SD	SE	N	Mean	±SD	SE			
Day 3	10	1.80	0.63	0.20	10	2.30	0.67	0.21	-1.709	0.105	NS
Day 5	10	2.40	0.52	0.16	10	2.80	0.79	0.25	-1.342	0.196	NS
Day 7	10	3.20	0.42	0.13	10	3.40	0.52	0.16	-0.949	0.355	NS
Day 14	10	3.80	0.63	0.20	10	4.20	0.63	0.20	-1.414	0.174	NS

When we compared the mean difference of the analgesics between Group 1 and Group 2, the mean score statistically differed from each other on days three and five, respectively, but the p-value was not calculated in the case of days seven and 14 because the mean difference is zero in this case (Table 7).

**Table 7:** Intergroup comparisons of analgesics Group I: Utilizing braided silk 3-0 sutures Group II: Using isoamyl-2 cyanoacrylate N: Number of patients, SD: Standard deviation, SE: Standard error, S: statistically significant A p-value was considered significant if p < 0.05. Test applied: Unpaired-t-test; NC stands for not calculated Analgesic comparison is for the kind of pain scale experienced by the patient.

Days	Group	N	Mean	±SD	SE	Un-paired t	P-value
Day-3	Group-1	10	5.300	1.418	0.448	3.334	0.004
	Group-2	10	3.200	1.398	0.442		
Day-5	Group-1	10	2.000	2.055	0.650	3.078	0.006
	Group-2	10	0.000	0.000	0.000		
Day-7	Group-1	10	0.000	0.000	0.000	NC	NC
	Group-2	10	0.000	0.000	0.000		
Day-14	Group-1	10	0.000	0.000	0.000	NC	NC
	Group-2	10	0.000	0.000	0.000		

The mean score of the intragroup comparison of analgesics in Group I statistically differs from each other at different time intervals. The mean score decreased from 5.300 on day three to 2.000 on day five, and it became 0.000 from day seven onwards, with a statistically significant difference of p = 0.001, as shown in Table 8.

**Table 8:** Intragroup comparisons of analgesics Group 1 Group I: Utilizing braided silk 3-0 sutures SD: Standard deviation, SE: Standard error Test applied: One-way analysis of variance (ANOVA) Significant: p < 0.05 Analgesic comparison is for the kind of pain scale experienced by the patient.

Time intervals	N	Mean	±SD	SE	F-ratio	P-value
Day 3	10	5.300	1.418	0.448	40.144	0.001
Day 5	10	2.000	2.055	0.650		
Day 7	10	0.000	0.000	0.000		
Day 14	10	0.000	0.000	0.000		

The mean score of the intragroup comparison of analgesics in Group II statistically differs from each other at different time intervals. The mean score worked out to be 3.200 on day three, and it was recorded to be 0.000 from day five onwards, with a statistically significant difference of p = 0.001, as shown in Table 9. 

**Table 9:** Intragroup comparisons of analgesics in Group 2 Group II: Using isoamyl-2 cyanoacrylate SD: Standard deviation, SE: Standard error Test applied: One-way analysis of variance (ANOVA) Significant: p < 0.05 Analgesic comparison is for the kind of pain scale experienced by the patient.

Time intervals	N	Mean	±SD	SE	F-ratio	P-value
Day 3	10	3.200	1.398	0.442	52.364	0.001
Day 5	10	0.000	0.000	0.000		
Day 7	10	0.000	0.000	0.000		
Day 14	10	0.000	0.000	0.000		

## Discussion

The main objective for the use of sutures in surgical procedures is to close the wound. This involves bringing together the edges of the incision so that the wound can heal properly. Over time, there have been advancements in the materials used for this purpose, specifically in the use of sutures [8-10]. The present study compared the use of iso-amyl 2-cyanoacrylate and conventional silk sutures after periodontal flap surgery in terms of patient compliance and wound healing.

The high risk of re-infection associated with the use of sutures, either due to biofilm accumulation or tissue trauma by syringes and needles, paved the way for research on alternative treatment options [11]. Cyanoacrylate tissue adhesive has been studied widely. The theory behind the action of cyanoacrylate is that alkyl 2-cyanoacrylates undergo an exothermic polymerization reaction catalyzed by the presence of small quantities of a weak base such as water. This action provides bonding action [12]. Acid stabilizers keep the glue in liquid form. The tissue adhesive has a hemostatic effect with a long half-life, good tissue tolerance, and gradual resorption with non-toxic actions [13]. The biological degradation of formaldehyde and cyanoacetate makes the product non-toxic [14].

The cases presented in the study, when evaluated statistically, depicted significant changes in intragroup comparison. The wound healing index significantly improved over the follow-up period. However, during the intergroup comparison, the values failed to reach a statistically significant result. Clinically, the duration of surgery, patient comfort, post-operative pain, and number of analgesics taken were less for the Group II patients with isoamyl 2-cyanoacrylate.

The study by Bhaskar et al. used cyanoacrylate on 235 patients. They recommended the use of cyanoacrylate adhesives as dressings after gingivectomy, flap surgery, extraction, and minor surgeries [15]. Kulkarni et al. conducted a clinical and histological study to compare healing after periodontal flap surgery. The results of their study depicted better healing in the cyanoacrylate group both clinically and histologically [5]. A similar study using the iso-amyl 2-cyanoacrylate group was done by Khurana et al. in 2016. They also concluded that similar results showed significant differences in intragroup comparisons. However, there was no significant difference in the intergroup comparison [16]. Sadatmansouri et al. in 2020, evaluated early healing using cyanoacrylate tissue adhesive. They found no statistically significant difference between sutures and the cyanoacrylate group. However, they concluded that patient compliance was better in the cyanoacrylate group. Therefore, it can be used as an alternative for wound closure [17]. In recent studies by Saquib et al., it was stated that cyanoacrylate has good bonding properties to hold the tissue margins together [18].

Even after being in practice for over a decade, the studies on the toxicity of cyanoacrylate are limited. A review on the toxicity of cyanoacrylate was conducted by Leggat et al., who reported that cyanoacrylate tissue adhesives pose occupational hazards for workers and are safer for patients. Excessive exposure may lead to respiratory and dermatological ailments [19].

Even though the results failed to reach a statistically significant level of comparison, patients in the cyanoacrylate group had better compliance, a shorter duration of surgery, and less post-operative pain as compared to the control group. The use of tissue adhesives also decreases the risk of cross-infection by needle injury or tissue penetration. The use of local anesthesia could also be reduced with the use of tissue adhesives, as they do not involve tissue penetration or tissue trauma.

Limitations of this study include its relatively small sample size and the use of a single institution for data collection, which may limit the generalizability of the findings to other populations or settings. Additionally, the study's duration may have been insufficient to capture long-term outcomes or potential complications associated with the two different methods of wound closure. Furthermore, the study's exclusion criteria, such as excluding patients with a smoking or tobacco consumption history, might limit the applicability of the results to a broader patient population. Finally, the study did not assess factors such as patient satisfaction or cost-effectiveness, which could provide additional insights into the clinical utility of cyanoacrylate tissue adhesive compared to traditional silk sutures.

## Conclusions

The present study concluded that the number of analgesics consumed post-operatively was lower in the cyanoacrylate group compared to the silk suture group. This suggests that isoamyl 2-cyanoacrylate can be used as an alternative to conventional silk sutures as it decreases post-operative pain and discomfort, shortens the duration of surgery, and initially has better wound healing properties. Further research with larger sample sizes and longer follow-up periods is warranted to confirm these findings and explore the long-term effects of cyanoacrylate used in periodontal surgery. Therefore, further investigation with larger cohorts and longer observation periods is necessary to confirm these preliminary results and thoroughly assess the long-term effects of cyanoacrylate in periodontal surgery.
